# Development and Validation of Cutoff Value for Reduced Muscle Mass for GLIM Criteria in Patients with Gastrointestinal and Hepatobiliary–Pancreatic Cancers

**DOI:** 10.3390/nu14050943

**Published:** 2022-02-23

**Authors:** Mami Takimoto, Sonoko Yasui-Yamada, Nanami Nasu, Natsumi Kagiya, Nozomi Aotani, Yumiko Kurokawa, Yoshiko Tani-Suzuki, Hideya Kashihara, Yu Saito, Masaaki Nishi, Mitsuo Shimada, Yasuhiro Hamada

**Affiliations:** 1 Department of Therapeutic Nutrition, Institute of Biomedical Sciences, Tokushima University Graduate School, 3-18-15 Kuramoto-cho, Tokushima 770-8503, Japan; mocchi.02110@gmail.com (M.T.); c202131004@tokushima-u.ac.jp (N.N.); kaginami@mirror.ocn.ne.jp (N.K.); aotani.nozomi@gmail.com (N.A.); kurokawa.yumiko@piano.ocn.ne.jp (Y.K.); suzuki-tani.yoshiko@tokushima-u.ac.jp (Y.T.-S.); saito.yu.1001@tokushima-u.ac.jp (Y.S.); hamada@tokushima-u.ac.jp (Y.H.); 2 Department of Digestive Surgery and Transplantation, Institute of Biomedical Sciences, Tokushima University Graduate School, 3-18-15 Kuramoto-cho, Tokushima 770-8503, Japan; kashihara.hideya@tokushima-u.ac.jp (H.K.); nishi.masaaki@tokushima-u.ac.jp (M.N.); mitsuo.shimada@tokushima-u.ac.jp (M.S.)

**Keywords:** Global Leadership Initiative on Malnutrition, reduced muscle mass, cutoff value, fat-free mass index, arm circumference, gastrointestinal cancer

## Abstract

The Global Leadership Initiative on Malnutrition (GLIM) criteria recommends using race- and sex-adjusted cutoff values for reduced muscle mass (RMM), but the only cutoff values available for Asians are the skeletal muscle mass index (SMI) established by the Asian Working Group for Sarcopenia (AWGS). This retrospective study aimed to develop and validate cutoff values for the fat-free mass index (FFMI) and arm circumference (AC) of Asians, and to investigate the association between GLIM malnutrition and prognosis. A total of 660 patients with primary gastrointestinal (GI) and hepatobiliary–pancreatic (HBP) cancers who underwent their first resection surgery were recruited and randomly divided into development and validation groups. The FFMI and AC cutoff values were calculated by receiver operating characteristic curve analysis for the AWGS SMI as the gold standard. The cutoff values for each RMM were used to diagnose malnutrition on the basis of GLIM criteria, and the survival rates were compared. The optimal FFMI cutoff values for RMM were 17 kg/m^2^ for men and 15 kg/m^2^ for women, and for AC were 27 cm for men and 25 cm for women. In the validation group, the accuracy of the FFMI and AC cutoff values to discriminate RMM were 85.2% and 68.8%, respectively. Using any of the three measures of RMM, overall survival rates were significantly lower in the GLIM malnutrition group. In conclusion, the cutoff values for the FFMI and AC in this study could discriminate RMM, and GLIM malnutrition using these cutoff values was associated with decreased survival.

## 1. Introduction

Malnutrition is common in patients with gastrointestinal (GI) and hepatobiliary–pancreatic (HBP) cancers; for example, the prevalence of malnutrition is reported to be 83%, 83%, and 60% in patients with pancreatic, gastric, and colorectal cancer, respectively [1]. Preoperative malnutrition has been associated with negative outcomes, including a prolonged hospital stay, increased postoperative complications, and high mortality [2,3,4]. Preoperative nutritional support has also been associated with a lower incidence of postoperative complications [5]. Therefore, early identification of malnourishment in patients is important to provide adequate nutritional support.

In 2018, the Global Leadership Initiative on Malnutrition (GLIM) criteria for diagnosis of malnutrition was published [6]. The purpose of this specific initiative was to reach a global consensus on the identification and endorsement of criteria for the diagnosis of malnutrition in clinical settings. The GLIM criteria propose a two-step model for risk screening and diagnosis assessment. The first step is to conduct nutritional screening using a validated screening tool. The second step is to assess three phenotypic and two etiologic criteria. Phenotypic criteria include non-intentional weight loss, low body mass index (BMI), and reduced muscle mass (RMM). Etiologic criteria include reduced food intake and assimilation as well as inflammation. Patients are diagnosed with malnutrition when at least one criterion from each phenotypic and etiologic component is present. For identifying RMM, the GLIM recommended the use of a fat-free mass index (FFMI) by dual-energy absorptiometry (DXA), bioelectrical impedance analysis (BIA), computed tomography (CT), and magnetic resonance imaging (MRI), but also recommended anthropometric measures as alternative measures when FFMI is not available. The GLIM recommends using race- and sex-adjusted cutoff values, but the only cutoff values available to Asians are the skeletal muscle mass index (SMI) established by the Asian Working Group for Sarcopenia (AWGS) [7]; cutoff values for other indicators such as FFMI and arm circumference (AC) remain unknown. The GLIM mentions that additional research is warranted to establish general reference standards as well as for some specific populations, e.g., in Asia [6].

This study aimed to (1) develop and validate cutoff values for the FFMI and AC relative to the cutoff value for the AWGS SMI as the gold standard, which would support the GLIM criteria for Asians, and (2) investigate the association between GLIM malnutrition and prognosis, when using the AWGS SMI cutoff values or calculated FFMI and AC cutoff values to identify RMM.

## 2. Materials and Methods

### 2.1. Patients

This is a retrospective, observational study. Patients admitted to the Department of Digestive Surgery and Transplantation in Tokushima University Hospital, Tokushima, Japan, from July 2014 to March 2021 were eligible for this study. The inclusion criteria were as follows: (1) Age 18 years or older, (2) diagnosed with primary GI and HBP cancers, and (3) determined to be eligible for radical resection and underwent first elective radical resection surgery. After screening for the inclusion criteria, 1029 patient records were collected. We excluded 18 patients with stage 0 or unknown stage, and 351 cases with missing data from the GLIM criteria assessment (missing data on weight loss [*n* = 6], BIA [*n* = 248], and AC [*n* = 97]). Finally, we analyzed the data of 660 patients. All patients were randomly divided into two groups, a development group and a validation group, so that the sex ratios were equal.

### 2.2. Data Collection

We collected the following data from the electronic medical records: age, sex, cancer site, cancer stage, surgical approach, preoperative therapy, adjuvant chemotherapy, height, and dates of operation and death.

### 2.3. Muscle Mass Measurements

All preoperative muscle mass measurements, including BIA and AC, were performed between admission and surgery by well-trained registered dietitians. BIA was performed using InBody 770 body composition and body water analyzer (InBody, Tokyo, Japan), and resistance and reactance were measured using an eight-point tactile electrode and multi-frequency current. Patients fasted for at least 4 h before the measurement. BIA was performed in a standing position and was not performed in patients with pacemakers or those who had difficulty standing. The InBody 770 automatically estimates fat-free mass (FFM) and appendicular skeletal muscle mass (ASM). The BMI was calculated as weight/height^2^ (kg/m^2^). The FFMI was calculated as FFM/height^2^ (kg/m^2^). The SMI was calculated as ASM/height^2^ (kg/m^2^). AC at the midpoint of the triceps of the non-dominant arm was measured with an insert tape.

### 2.4. Development of Cutoff Values for the FFMI and AC

In the development group, receiver operating characteristic (ROC) curve analysis was performed to calculate the cutoff values for the FFMI and AC to discriminate the cutoff value for the SMI according to AWGS. The optimal cutoff values for the FFMI and AC were determined based on the nearest ROC curve point to the corner of both sensitivity and specificity of 1.0.

### 2.5. Validation of Cutoff Values for the Calculated FFMI and AC

In the validation group, the cutoff values for the FFMI and AC calculated in the development group were validated. First, the sensitivity, specificity, positive predictive value (PPV), negative predictive value (NPV), and accuracy of the developed cutoff values for the FFMI and AC were calculated based on the SMI of AWGS. Second, the patients were diagnosed with malnutrition according to the GLIM criteria to compare the prevalence of GLIM malnutrition when using the SMI, FFMI, and AC. According to the first step in diagnosing GLIM malnutrition, the risk of malnutrition was screened using the Malnutrition Screening Tool (MST) [8]. Patients considered to be “at risk for malnutrition” with an MST score of 2 or higher proceeded to the next step. The second step involved diagnosing malnutrition on the basis of three phenotypic criteria ((a) unintentional weight loss; >5% within past 6 months, or >10% beyond 6 months; (b) low BMI for Asians; <18.5 kg/m^2^ if age < 70 years, or <20 kg/m^2^ if age ≥ 70 years; (c) RMM) and two etiologic criteria ((d) reduced food intake or assimilation; (e) inflammation). At least one criterion from each phenotypic and etiologic criterion was necessary for a malnutrition diagnosis. All patients were considered to fit the criterion for inflammation because all subjects in this study were patients with cancer. The sensitivity, specificity, PPV, NPV, and accuracy of the GLIM using FFMI or AC were calculated based on the GLIM using SMI.

### 2.6. Survival Analysis

The mortality was compared between GLIM malnutrition and non-malnutrition when using the SMI, FFMI, and AC in all patients. We calculated the time of surgery to the last follow-up date (31 July 2021) or death.

### 2.7. Statistical Analysis

Continuous variables with non-normal distributions were presented as the median and interquartile range. Comparisons of continuous variables between the development group and validation group were analyzed by Wilcoxon’s rank sum test. Comparisons of categorical variables between the two groups were analyzed using the chi-squared test. In all patients, survival curves were plotted using the Kaplan–Meier method and differences were evaluated using the log-rank test. ROC curve analysis was performed to calculate the cutoff value for the FFMI and AC to predict death within 3 years. All statistical analyses were performed using JMP ver. 13.0 (SAS Institute, Cary, NC, USA). All *p*-values < 0.05 were considered statistically significant.

## 3. Results

Table 1 presents the characteristics of the 660 patients who were analyzed in this study. There were no significantly different variables between the development and validation groups.

**Figure 1:**
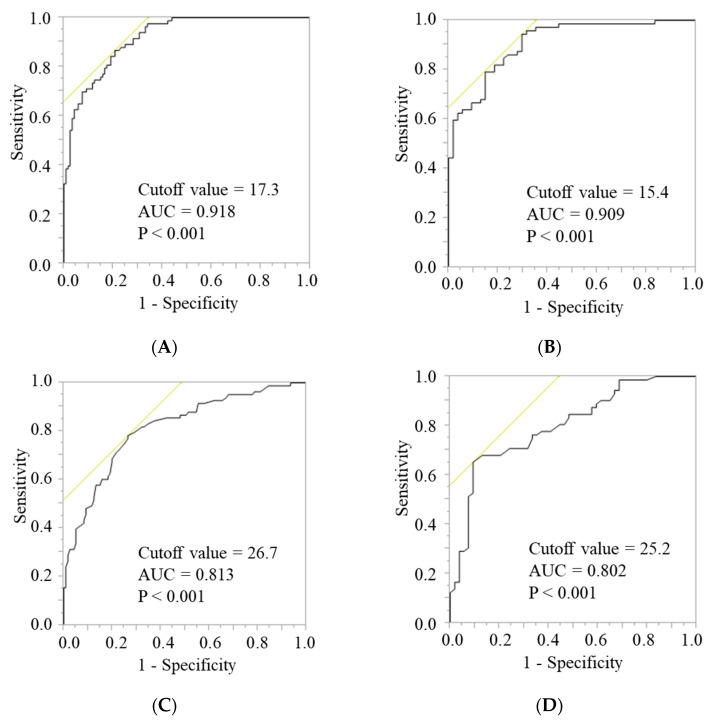
ROC curves to determine the cutoff values for the FFMI and AC, which discriminate the cutoff value for the SMI according to AWGS in the development group. (**A**) FFMI in men; (**B**) FFMI in women; (**C**) AC in men; (**D**) AC in women. ROC, receiver operating characteristics; FFMI, fat-free mass index; AC, arm circumference; SMI, skeletal muscle mass index; AWGS, Asian Working Group for Sarcopenia; AUC, area under the curve.

In the development group, 330 patients (204 men and 126 women) were analyzed to calculate the cutoff values for the FFMI and AC according to sex. ROC analyses showed that an FFMI of 17.3 kg/m^2^ for men and 15.4 kg/m^2^ for women, and an AC of 26.7 cm for men and 25.2 cm for women were the optimal cutoff points for discriminating the SMI cutoff value for AWGS, which were statistically significant (Figure 1). We used the rounded cutoff values for subsequent analyses (FFMI < 17 kg/m^2^ in men, FFMI < 15 kg/m^2^ in women; AC < 27 cm in men, and AC < 25 cm in women).

In the validation group, 330 patients (203 men, 127 women) were analyzed to validate the developed cutoff values for the FFMI and AC. The percentage of low SMI (SMI < 7.0 kg/m^2^ in men, SMI < 5.7 kg/m^2^ in women) was 40.9% in men and 58.3% in women. The ratio of low FFMI (FFMI < 17 kg/m^2^ in men, FFMI < 15 kg/m^2^ in women) was 40.9% in men and 44.9% in women and the ratio of low AC (AC < 27 cm in men, and AC < 25 cm in women) was 53.2% in men and 33.1% in women. The developed FFMI and AC cutoff values were statistically compared with the SMI cutoff value for AWGS (Table 2). The sensitivity and specificity of FFMI were 79.0% and 90.8% and of AC were 65.0% and 72.3%, respectively.

Next, 330 patients in the validation group were diagnosed with GLIM malnutrition using the AWGS SMI, developed FFMI, or developed AC as the indicator of RMM. The prevalence of GLIM malnutrition was 22.4% when using the SMI, 20.9% when using the FFMI, and 21.2% when using AC. Statistical evaluations are shown in Table 3. The sensitivity and specificity of GLIM using the FFMI were 93.2% and 100.0% and using AC were 91.9% and 99.2%, respectively.

**Figure 2:**
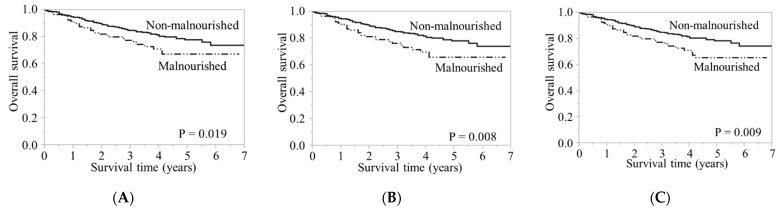
Kaplan–Meier survival curves by GLIM criteria malnutrition. (**A**) GLIM using SMI; (**B**) GLIM using FFMI; (**C**) GLIM using AC in all patients. GLIM, Global Leadership Initiative on Malnutrition; SMI, skeletal muscle mass index; FFMI, fat-free mass index; AC, arm circumference.

Figure 2 shows the survival curves of the malnourished and non-malnourished group according to the GLIM criteria in all patients. In the malnourished group, the five-year survival rate was 68%, 66%, and 66% when using the SMI, FFMI, and AC, respectively. In the non-malnourished group, the five-year survival rate was 78%, 78%, and 79% when using the SMI, FFMI, and AC, respectively. GLIM malnutrition had a significantly poor prognosis when any of the three indicators of RMM was used.

Finally, 374 patients who could follow-up for three years were analyzed for ROC curve analysis to calculate the cutoff values for the FFMI and AC to predict death within three years. From the results of the ROC curve analysis, the FFMI of 17.0 kg/m^2^ for men (area under the curve (AUC) = 0.673, *p* < 0.001) and 15.0 kg/m^2^ for women (AUC = 0.657, *p* = 0.010), and AC of 26.2 cm for men (AUC = 0.720, *p* < 0.001) and 25.3 cm for women (AUC = 0.675, *p* = 0.004) were determined to be the statistically significant cutoff values for predicting three-year mortality.

## 4. Discussion

The present study investigated the cutoff values for the FFMI and AC as indexes of the RMM available for Asian populations, with AWGS SMI cutoff values utilized as the gold standard. The optimal FFMI cutoff values for RMM were 17 kg/m^2^ for men and 15 kg/m^2^ for women. The optimal AC cutoff values for RMM were 27 cm for men and 25 cm for women. Our results showed that patients diagnosed with GLIM malnutrition had lower survival rates than non-malnutrition patients.

In this study, we focused on the FFMI and AC because the FFMI has been proposed as a useful alternative measure of the RMM [6], and AC has been widely used in clinical settings because of its low cost and availability. It was reported that AC was positively correlated with the SMI calculated by DXA [9]. It was reported that there are race-related differences in the values for the SMI, FFMI, and AC, which are lower in Asian populations than in Western populations, when compared among people of the same ages of each sex [10,11,12,13]. Therefore, the cutoff value for the RMM must be calculated for Asian demographics [6,10].

Recently, a Chinese study reported that the optimal AC cutoff values for a low SMI based on AWGS2019 were 28.6 cm for men and 27.5 cm for women [14], which were higher than the values determined by our study. This may have been due to differences in the technique used for taking measurements: in the Chinese study, the measurement was performed with the dominant hand, whereas in the present study, the measurement was performed with the non-dominant hand. While the Chinese report only proposed an AC cutoff value to discriminate a low SMI, this study found that the criterion for GLIM malnutrition using the obtained AC cutoff value showed poor prognosis. In addition, the present study challenged to calculate the optimal cutoff value for the FFMI and AC to predict three-year survival. As a result, we obtained a cutoff value for the FFMI (17.0 kg/m^2^ for men and 15.0 kg/m^2^ for women) and AC (26.2 cm for men and 25.3 cm for women). These were close to the cutoff values determined by our results for identifying the AWGS2019 SMI (FFMI: 17 kg/m^2^ for men, 15 kg/m^2^ for women; AC: 27 cm for men, 25 cm for women). These results indicated that the cutoff values for the FFMI (17 kg/m^2^ for men and 15 kg/m^2^ for women) and AC (27 cm for men and 25 cm for women) could not only discriminate the RMM but also predict prognosis. This is the first report that was able to propose cutoff values for the FFMI and AC, which discriminate the RMM and predict the prognosis for Asian patients. We believe that this study is meaningful because AWGS 2014 mentioned that due to a lack of outcome-based data, the classical approach (i.e., below two standard deviations of the mean muscle mass of a young reference group, or the lower quintile) was proposed for determining the cutoff value for the SMI [15].

The cutoff values for the FFMI and AC calculated in this study were close to the median values of healthy Japanese of the same age [16,17]. These results indicated that approximately half of the elderly Japanese population will be considered to have RMM according to the FFMI and AC cutoff values calculated in this study. Previous systematic reviews and meta-analyses reported that nutritional interventions or the combination of nutritional interventions and strength exercise showed a significantly positive effect on muscle mass in the elderly population [18,19]. Further studies are required to examine whether increasing muscle mass by these interventions leads to improved prognosis.

According to the GLIM criteria, the rate of malnutrition was approximately 20% using any of the three indicators of RMM (SMI, FFMI, and AC). The three indicators of RMM were considered to have the same level of capability for determination. In a previous study using calf circumference as a measure of muscle mass, 18.0% of Japanese hospitalized patients were malnourished based on GLIM criteria [20]. The study on patients with gastrointestinal cancer showed that 27.3% were malnourished when the CT-derived L3 SMI was used as a measure of muscle mass [21]. The prevalence of malnutrition in the present study was similar to these previous reports.

It was reported that the GLIM criteria without RMM were less sensitive than the GLIM criteria with RMM [22,23]. Wang Y et al. revealed that any use of an indicator of RMM (calf circumference, FFMI, and SMI) increased the sensitivity of diagnosing malnutrition compared with GLIM criteria that excluded RMM, which yielded results that were in better accordance with PG-SGA [23]. This indicated the importance of muscle mass evaluation, even when using simple methods. Recent literature for cancer patients has shown that patients with a low FFMI based on the BIA had shorter survival and poorer quality of life compared to patients with a normal FFMI [24]. Wang Y et al. argued that there is strong evidence to support the inclusion of RMM as one of the three phenotypic criteria of the GLIM criteria [23].

Previous studies reported the association between GLIM-defined malnutrition and survival [22,25,26,27,28,29,30,31,32], length of hospital stay [29], and postoperative pulmonary complication [25]. Our study showed that, regarding patients who had GI and HBP cancers, patients who were malnourished according to the GLIM criteria were associated with a shorter survival compared to patients who were non-malnourished. This result was consistent with previous reports. Although the data are not shown, we had performed subgroup analyses according to cancer site and stage. Regarding the cancer site, similar results were obtained for gastric and colorectal cancers, showing that GLIM malnutrition was associated with a poorer prognosis than non-malnutrition. Regarding the cancer stage, similar results were obtained only for Stage III. However, the results of these subgroup analyses were preliminary due to the small sample size. Further studies are therefore needed to clarify whether GLIM malnutrition using the developed cutoff value for FFMI and AC can distinguish the prognosis of patients with different tumors and at different stages. The relationship between malnutrition and death has long been known. The loss of lean body mass leads to impaired immune response and organ function, which results in death due to protein depletion [33].

The strength of the present study is that this is the first report to propose cutoff values for RMM specifically in Asians, which could predict prognoses. However, this study had some limitations. First, this study was a single center study, and the population consisted only of hospitalized patients with GI and HBP cancers. Therefore, our findings may not be generalizable to other diseases or community-dwelling populations. Further multi-center studies involving other populations are required to validate our results. Second, the number of excluded patients was large because of data missing for diagnosing malnutrition based on GLIM criteria, which might have affected the results. Third, our results on the association between GLIM malnutrition and prognosis did not account for the cancer site, cancer stage, type of surgery, and the presence of preoperative therapy or adjuvant chemotherapy. Further studies with a larger sample size are thus required.

## 5. Conclusions

This study reported the cutoff values for the FFMI (men < 17 kg/m^2^, women < 15 kg/m^2^) and AC (men < 27 cm, women < 25 cm), which discriminate the RMM and predict survival in Asian patients with GI and HBP cancers to evaluate potential race- and sex-adjusted cutoff values. Malnutrition based on GLIM criteria was associated with decreased survival. Further studies are required to clarify the outcome-based cutoff value for RMM.

## Figures and Tables

**Table 1:** Patient characteristics.

	All	Development	Validation	*p*-Value
	*n* = 660	*n* = 330	*n* = 330	
Age (years)	70 (63–76)	70 (64–76)	70 (63–76)	0.572
Sex (*n*, %)				0.936
Men	407 (61.7)	204 (61.8)	203 (61.5)	
Women	253 (38.3)	126 (38.2)	127 (38.5)	
Cancer site (*n*, %)				0.686
Colorectal	259 (39.2)	137 (41.5)	122 (37.0)	
Stomach	188 (28.5)	86 (26.1)	102 (30.9)	
Liver	81 (12.3)	41 (12.4)	40 (12.1)	
Bile duct	59 (8.9)	30 (9.1)	29 (8.8)	
Pancreas	73 (11.1)	36 (10.9)	37 (11.2)	
Stage (*n*, %)				0.169
I	206 (31.2)	106 (32.1)	100 (30.3)	
II	236 (35.8)	116 (35.2)	120 (36.4)	
III	156 (23.6)	70 (21.2)	86 (26.1)	
IV	62 (9.4)	38 (11.5)	24 (7.3)	
Surgical approach (*n*, %)				0.876
Laparotomy	296 (44.9)	147 (44.6)	149 (45.2)	
Laparoscopic surgery	364 (55.2)	183 (55.5)	181 (54.9)	
Preoperative therapy (*n*, %)	73 (11.1)	35 (10.6)	38 (11.5)	0.710
Adjuvant chemotherapy (*n*, %)	249 (37.7)	127 (38.5)	122 (37.0)	0.688
Height (cm)	160.0 (152.0–167.0)	160.0 (152.0–167.1)	160.0 (151.8–167.0)	0.649
Body weight (kg)	56.8 (49.0–64.4)	57.2 (49.7–65.6)	56.3 (48.7–63.7)	0.305
BMI (kg/m^2^)	22.3 (20.3–24.2)	22.4 (20.5–24.4)	22.2 (20.1–24.0)	0.289
SMI (kg/m^2^)	6.6 (5.7–7.4)	6.7 (5.7–7.3)	6.5 (5.7–7.4)	0.595
Low SMI * (*n*, %)	312 (47.3)	155 (47.0)	157 (47.6)	0.876
FFMI (kg/m^2^)	16.4 (15.1–17.9)	16.6 (15.1–17.9)	16.3 (15.2–18.0)	0.712
AC (cm)	26.6 (24.6–28.6)	26.6 (24.6–28.6)	26.7 (24.8–28.4)	0.999

* Low SMI was defined as an SMI < 7.0 kg/m^2^ in men and SMI < 5.7 kg/m^2^ in women according to the cutoff value for AWGS. BMI, body mass index; SMI, skeletal muscle mass index; FFMI, fat-free mass index; AC, arm circumference; AWGS, Asian Working Group for Sarcopenia.

**Table 2:** Statistical evaluations of developed cutoff values for the FFMI and AC to discriminate the cutoff value for the SMI according to AWGS * in the validation group.

	FFMI **	AC ^†^
Sensitivity (%)	79.0	65.0
Specificity (%)	90.8	72.3
PPV (%)	88.6	68.0
NPV (%)	82.6	69.4
Accuracy (%)	85.2	68.8

* SMI < 7.0 kg/m^2^ in men, SMI < 5.7 kg/m^2^ in women. ** FFMI < 17 kg/m^2^ in men, <15 kg/m^2^ in women. ^†^ AC < 27 cm in men, <25 cm in women. FFMI, fat-free mass index; AC, arm circumference; SMI, skeletal muscle mass index; AWGS, Asian Working Group for Sarcopenia; PPV, positive predictive value; NPV, negative predictive value.

**Table 3:** Statistical evaluations of GLIM malnutrition using FFMI or AC for GLIM malnutrition using the SMI * in the validation group.

	GLIM Using FFMI **	GLIM Using AC ^†^
Sensitivity (%)	93.2	91.9
Specificity (%)	100.0	99.2
PPV (%)	100.0	97.1
NPV (%)	98.1	97.7
Accuracy (%)	98.5	97.6

* SMI < 7.0 kg/m^2^ in men, SMI < 5.7 kg/m^2^ in women. ** FFMI < 17 kg/m^2^ in men, <15 kg/m^2^ in women. ^†^ AC < 27 cm in men, <25 cm in women. GLIM, Global Leadership Initiative on Malnutrition; FFMI, fat-free mass index; AC, arm circumference; SMI, skeletal muscle mass index; PPV, positive predictive value; NPV, negative predictive value.

## Data Availability

The data presented in this study are available upon reasonable request from the corresponding author following permission by the Ethics Committee and the hospital at which the study was conducted.
